# Editorial: The Plant Holobiont Volume I: Microbiota as Part of the Holobiont; Challenges for Agriculture

**DOI:** 10.3389/fpls.2021.799168

**Published:** 2021-11-30

**Authors:** Patrizia Cesaro, Elisa Gamalero, Junling Zhang, Barbara Pivato

**Affiliations:** ^1^Dipartimento di Scienze e Innovazione Tecnologica, Università del Piemonte Orientale, Alessandria, Italy; ^2^Key Laboratory of Plant–Soil Interactions, Ministry of Education, College of Resources and Environmental Sciences, China Agricultural University, Beijing, China; ^3^Agroécologie, AgroSup Dijon, INRAE, Univ. Bourgogne, Univ. Bourgogne Franche-Comté, Dijon, France

**Keywords:** holobiont, rhizosphere microbiome, biotic/abiotic stress, beneficial microorganisms, root exudates, plant-microbe interactions, agricultural practices

## Introduction

A possible approach to ensure the sustainability of cropping systems in agroecology is to promote beneficial interactions between plants and their associated microbiota. Plant-microbe interactions in the rhizosphere are often described as a feedback-loop in which plants recruit, through the release of exudates, specific rhizosphere microbiota that, in turn, affect plant growth and health. Furthermore, these interactions can change during the different phases of the life cycle of the organisms.

Recently, the concept of holobiont has been introduced for crop plants. The term holobiont was first defined by Margulis (1991), as a simple biological unit involving a host and a single inherited symbiont. Then, Zilber-Rosenberg and Rosenberg (2008) have expanded this definition to the entire microbiota and proposed the holobiont to be a selection unit, which underlies the hologenome based theory of evolution.

The added value of the hologenomic view of crop-microbe interactions in agroecology is to better take into account plant-microbe interactions and their results on plant biology, ecology and evolution. This represents a major issue in the conception of the so-called plant ideotype, defined as a plant genotype chosen for its capacity to better exploit a given environment and to adapt to abiotic and biotic stresses.

The next step will be to consider interactions between plants within a plant canopy (mono- or pluri-species) as interactions between holobionts when taking into account ecosystem services expected from agroecosystems (e.g., food production, climate mitigation, water biofiltration).

Thus, new plant genotypes need to be selected in order to better value rhizodeposits and plant-microbiota interactions in the rhizosphere. This will require the identification of plant traits involved in the recruitment of beneficial microbial populations/functions.

For reaching that target, further research is needed to (i) identify plant and microbiota determinants for a beneficial interaction, (ii) define functional crop holobionts, (iii) design cropping systems that value the best the corresponding holobionts, and (iv) understand and predict the eco-evolution of the beneficial plant-microbe interactions.

We host in this Research Topic, “The Plant Holobiont Volume I: Microbiota as Part of the Holobiont; Challenges for Agriculture,” 14 articles (12 research articles, 1 method article and 1 review article) enhancing our knowledge on agricultural practices (organic and conventional farming, intercropping) and plant traits (exudates, genes) modulating rhizosphere microbiota (bacterial and fungal communities) under different environments and biotic (e.g., *Rhizoctonia solani*) and abiotic (i.e., drought, heavy metal) stresses. On the other way round, the beneficial effect of some rhizospheric strains on plant growth and health, and the transmission of these beneficial strains from one plant generation to the following are also described. Sixteen plant species were used to perform these studies: 10 crop plants (grapevine, four leafy green crops, maize, pea, pumpkin, rice, sugar beet, sugarcane, and wheat), two trees (olive and poplar), a model (*Brachypodium distachion*), and a grass (ryegrass). Some of these studies characterized the impact of different genotypes/cultivars (maize, pea, pumpkin, rice, sugar beet, and wheat) on the microbiota and one (Zhao et al.) assessed the impact of a mutant plant. Finally, the study of Zhou et al. made a first attempt to correlate microbial community composition to grapevine berries and wine properties.

## Content Collection

### Original Research Articles

Several articles confirmed the well-known plant species effect on rhizosphere microbiota. This rhizosphere effect was shown to be modulated by the soil type when considering saprophytic and symbiotic fungi (Zhou et al.) and by the plant species (Cesaro et al.; Pivato et al.), but did not differ upon the developmental stage of grapevine (Cesaro et al.).

Beyond soil physico-chemical parameters and plant species, the farming system deeply affects rhizosphere microbiota. Bacterial diversity in maize rhizosphere was higher when cultivated in organic than in conventional farming (Ares et al.). Organic amendment increased only the bacterial but not the fungal diversity in poplar rhizosphere; however, this greater microbiome diversity does not imply a better plant wellness or phytoremediation ability in zinc-polluted soil (Guarino et al.).

Intercropping agricultural practice tested by Pivato et al. affected the co-occurrence network of rhizosphere bacterial communities but not their diversity. However, the variations of the microbiota (diversity and network of co-occurrence) mediated by the agricultural practices on the host-plant mostly remain untapped and further studies are needed to elucidate these effects in order to identify the practices that allow to better value plant-microbe interactions.

Beside agricultural practices, the impact of the plant genotypes/cultivars on rhizosphere microbiota was also evaluated. Two maize genotypes (“Pigarro,” improved landrace and “SinPre,” a composite cross population), did not affect bacterial microbiota, although they shared a core bacteriome (Ares et al.). Chang et al. characterized the impact of 12 rice accessions (five wild and seven domesticated) on the diversity, structure and co-occurrence network of fungal communities. Domestication increased the alpha-diversity of the rice rhizosphere fungal communities, however, the co-occurrence network of symbiotic and saprophytic fungi was more developed in the wild rice rhizosphere. Pivato et al. showed differences in the bacterial diversity between pea hr and Hr genotypes, whilst no differences were found between wheat genotypes. The impact of plant genotype/cultivar on microbiota seems not to be consistent and to vary according plant genotypes/cultivars. Further research will have to understand the rationale for these variations.

Some works hosted in this Research Topic made attempts to identify plant genotypes able to select beneficial microbiota. Dumigan et al. characterized the impact of the wild maize ancestor *Zea mays* ssp. *parviglumis*, and an ancient mexican landrace (*Z. mays* spp. *mays*) on diazotrophs endophytes. The authors identified four strains able to growth in N-free media and to increase annual ryegrass biomass, that seem to come from the seeds and not from the soil. By using both molecular and culturomic approaches, Taffner et al. could characterize the diversity of bacterial communities of four indigenous leafy green vegetables but also their antagonistic activities against phytopathogenic fungi. A large core microbiome common in the four plants comprising 18 prokaryotic families was found, and the bacterial culture approach allowed to identify Sphingomonadaceae and Bacillaceae as key candidates for sustainable biocontrol agents. Wolfgang et al. used five different cultivars of sugar beet in order to characterize microbial populations, which are involved in plant protection against *R. solani*. Three bacterial genera preferentially associated to *Rhizoctonia*-tolerant cultivars are expected to contribute to this tolerance. Taken together, these results show that cultivars that are tolerant to this soilborne disease host microbial communities that account for the low disease expression, thus clearly supporting the reciprocal beneficial effect of plants and associated microbiota.

The legacy effect of the rhizosphere effect has been further explored by testing the possible transmission of rhizosphere microbiota (fungi and bacteria) of *Cucurbita pepo* to their progeny seeds (Kusstatscher et al.). Beneficial bacterial taxa appeared to be enriched in progeny seeds, indicating that beneficial microorganisms recruited from soil may be transmitted to the following plant generation.

However, if plants actively recruit beneficial microbiota, many aspects of this process remain to be untapped. Two articles in this Research Topic focused on the identification of plant traits mediating the recruitment and activities of beneficial microbial populations. Mavrodi et al. identified plant phenotypic traits that modulate microbial functioning. Characterization of the RNA-seq profiling of *Pseudomonas* cultures in the presence of root exudates of *Brachypodium dystachyon* stressed the impact of these exudates on the expression of genes encoding numerous catabolic and anabolic enzymes, transporters, transcriptional regulators, stress response, and conserved hypothetical proteins. Zhao et al. described the fungal community of the transgenic (TG) sugarcane variety GN18, harboring the drought-tolerant gene *Ea-DREB2B* and its corresponding non-TG wild-type (WT) variety, FN95-1702, suggesting this gene as plant determinant able to modulate the microbial community. These studies open future perspectives to identify plant determinants structuring rhizospheric communities.

Finally, rhizospheric microbiota on plant were reported to not only promote growth and health, but also nutritional quality of edible parts. A possible relation between bacterial community and fruit quality was assessed (Zhou et al.). The strategy followed consisted in comparing fruit/wine quality and bacterial communities from 22 different sites. However, differences in wine quality between these sites not only rely on the bacteriome but also on a series of parameters involved in the so-called “terroir.”

### Method Article

Articles in the Research Topic used amplicon metagenomics sequencing to characterize genetic diversity and structure of microbial communities and culturomic (sometimes coupled with genomics, as in Mavrodi et al.) to characterize their functionality. However, even if NGS are widely used, developments still need to be made specially when targeting plant niches. Haro et al. assessed the influence of commercially available DNA extraction kits and different primer pairs to produce a non-biased version of the composition of bacterial communities present in olive xylem sap. They showed a most accurate depiction of a bacterial mock community artificially inoculated on sap samples when using the PowerPlant DNA extraction kit, the combination of 799/1193R primers amplifying the hypervariable V5-V7 region and the Silva 132 database for taxonomic assignment. Moreover, Li et al. focused the first part of their review article on methodological approaches in order to characterize orchid mycorrhizal fungi and they identified the method that they consider as the most efficient. Progress is constantly been made also in bioinformatics analysis; for instance the use of Amplicon Sequence Variants (ASV, here used in Wolfgang et al.) instead of OTUs allows a better control of DNA extraction and PCR bias (Callahan et al., 2017).

### Review Articles

Li et al. concerns in mycorrizal diversity in Orchids made the only review article of the Research Topic. Orchids have complex symbiotic relationships with fungi at various stages of their life cycle. Environmental conditions significantly affect Orchid Mycorrhizal Fungi (OMF) diversity and abundance and plant deterministic processes seem to contribute to the construction of OMF communities. Methodological improvement and standardization could facilitate better comparability of core fungal taxa of different or the same orchid species from different case studies. Clarifying the architecture of the OMF networks formed by co-existing orchids can help to comprehend how these hyper-diverse interacting guilds are maintained and co-evolve in their habitats. This can further reveal the species, lineages, or functional taxa that are significant to ecosystem services.

## Author Contributions

All authors listed have made a substantial, direct, and intellectual contribution to the work and approved it for publication.

## Funding

BP was supported by Plant2Pro-Carnot Institute “POSiTiF” project.

## Conflict of Interest

The authors declare that the research was conducted in the absence of any commercial or financial relationships that could be construed as a potential conflict of interest.

## Publisher's Note

All claims expressed in this article are solely those of the authors and do not necessarily represent those of their affiliated organizations, or those of the publisher, the editors and the reviewers. Any product that may be evaluated in this article, or claim that may be made by its manufacturer, is not guaranteed or endorsed by the publisher.
